# A national analysis of the medical schools of training for merit award-winning laboratory medical doctors working in Britain

**DOI:** 10.1186/s12909-023-04161-z

**Published:** 2023-04-07

**Authors:** S. Steele, G. Andrade

**Affiliations:** Ajman University, Ajman, UAE

**Keywords:** Medical training, Medical careers, Medical schools, Award-winning, Britain, Career success

## Abstract

**Aims:**

To inform the discussion regarding the origins of Laboratory Medical Consultant clinical merit award holders (LMC) whether the awards came from the Clinical Excellence Awards (CEA) or Distinction Awards (DA) schemes.

**Methods:**

Setting - CEA is a scheme to financially reward senior doctors in England and Wales who are assessed to be working over and above the standard expected of their role. The DA scheme is the parallel and equivalent scheme in Scotland. Participants - All of the merit award holders in the 2019 round. Design - This involved a secondary analysis of the complete 2019 published dataset of award winners. Statistical analyses were performed with Chi-square tests set at p < 0.05 level for statistical significance.

**Results:**

The top five medical schools (London University, Glasgow, Edinburgh, Aberdeen and Oxford) were responsible for 68.4% of the LMC merit award holders in the 2019 round. 97.9% of the LMC merit award holders were from European medical schools, whereas 90.9% of the non-LMC award holders were from European medical schools. The LMCs with A plus or platinum awards came from only six medical schools: Aberdeen, Edinburgh, London University, Oxford, Sheffield and Southampton. In contrast, the B or silver/bronze LMC award holders came from a more diverse background of 13 medical schools.

**Conclusions:**

The majority of LMC merit award holders originated from only five university medical schools. All the LMCs with A plus or platinum awards came from only six university medical schools. There is an apparent overrepresentation of a small number of medical schools of origin amongst those LMCs that hold national merit awards.

## Background

The backbone of any good clinical medical practice is the quality of the laboratory medical doctors that support the frontline clinicians. These laboratory medical consultants (LMC) include consultants in the disciplines of pathology, haematology, biochemistry, clinical genetics, microbiology and immunology. Our project examines the medical schools of origin of these award-winning LMC clinicians.

In Britain, there are currently two national merit award schemes in operation to reward senior clinicians working in the National Health Service (NHS), the Clinical Excellence Awards Scheme (covering England and Wales) and the Distinction Awards Scheme (covering Scotland). [1] The doctors receiving such awards benefit not only from the positive career and reputational effects but also because they are the main explicitly financial incentive schemes for consultants. [1] The excellence awards are designed to reward all domains of clinical excellence including providing a high quality service, developing a high quality service, leadership and managing a high quality service, research and innovation, and teaching and training. The cost of these clinical excellence awards is substantial; in 2019-20 2061 such awards were given of which 300 were new award holders with a total cost of £125,801,942. [2] In 2016-17 the recipient clinicians annually received approximately £35,832 for bronze, £47,110 for silver, £58,888 for gold and £76,554 for platinum awards. National CEAs at all levels remain pensionable and are held for five years as long as clinicians remain eligible. The Scottish Distinction Awards are also paid in a similarly lucrative sliding scale and are also renewable every five years; their scheme, however, has been frozen in terms of the number of new awards since 2010.

The award schemes were established in the post-World War II era with a view to encouraging senior doctors to support the newly-formed NHS. However, the awards themselves have been the source of much discussion. Accordingly, they have been analyzed and discussed with regard to award objectivity, [3–6] distribution by specialty, [1, 7] by economic efficiency, [8] by region, [7] by gender, [1, 9–11] by age [8] and by ethnicity. [1, 12, 13] Nonetheless many commentators agree that some system should be in place to reward successful consultants. [14] This study aims to add to the discussion by relating the LMC and non-LMC award holders to their medical schools of origin. We place our findings in the context of educational, demographic and career implications for doctors aspiring to gain merit awards.

## Materials and methods

The names of the consultant and senior clinician merit award holders in laboratory medicine, were obtained from the Distinction Awards Annual Report, 2020 [15] and the Clinical Excellence Awards Report, 2020 [16] for the 2019 round. The medical schools of origin were identified with the UK Medical Register [17] and the UK Dental Register. [18] The total number of merit award holders was 901; the medical school of origin was identified for 99.8% of these doctors. Consequently, 899 participants were included in the dataset. Any award holding doctors in the above publications designated as laboratory medical doctors or histopathologists, general pathologists, forensic pathologists, molecular pathologists, haematopathologists or haematologists, transfusion specialists, biochemists or chemical pathologists, (clinical) geneticists, microbiologists/infectious disease consultants or immunologists were included as LMCs in this study.

The rankings of merit award holding medical schools were determined by summation of the number of LMC award holders of A plus, A or B grade (or platinum, gold or silver/bronze award holders). Combining these parallel and similar award gradings permitted all of Britain’s (England, Wales and Scotland) excellence award-winners to be analyzed together. Only these national level Clinical Excellence Awards and Distinction Awards were included in this study. As part of our analysis of the grades of awards we combined the award categories to explicitly show the three tiers of national merit awards; A plus and platinum award holders were combined to yield the top tier (tier 1) of national awards. The A and gold awards were combined to create the intermediate tier (tier 2) of national awards. Finally, the B and silver/bronze awards were combined to create the lowest tier (tier 3) of national merit awards. The same approach was taken with the non-LMC data.

The rankings of the merit award holding medical schools were approximately size-corrected by dividing the number of award holders that were graduates of the medical school by the number of admissions to the undergraduate medical school in the 2019-20 academic year. We used this pragmatic approach to estimate the size correction rather than the ideal but inaccessible integral of medical school graduation numbers against time for approximately the last 50 years.

The comparisons of distributions of award holders between LMCs and non-LMCs were quantified using Pearson’s Chi-Square test with the significance level set to p < 0.05 level.

In order to evaluate the international medical graduates we designated UK and Irish medical schools as local institutions and so were able to identify international medical graduates (IMGs).

All procedures were performed in accordance with relevant guidelines.

## Results

Our data indicated that the largest proportion of LMC award holders were pathologists at 64% of all the LMC specialist award holders.

Table 1 shows the ten medical schools that attained the greatest number of merit award winners; these award holders possessed tier 1, tier 2 or tier 3 awards. Table 1 also compares the medical schools of origin of LMC and non-LMC merit award holders for the ten medical schools with the greatest numbers of award holders; the table contrasts the proportions of LMC award holders and non-LMC award holders that the graduates of each medical school achieved. Pearson’s Chi-Square test showed no statistically significant difference between the distributions for the medical schools of origin for LMCs and non-LMCs, p > 0.05. Graduates of London, Glasgow, Edinburgh, Aberdeen and Oxford medical schools accounted for 68.4% of all national merit awards held by LMCs. The ten university medical schools in Table 1 accounted for 82.1% of all the LMC national merit award holders.

Table 2 demonstrates the effect of the approximate size correction for medical school size on the ranking of the medical schools of origin for the LMCs. Aberdeen and Glasgow medical schools remain first and second ranked respectively even after the approximate size correction is applied.

Our analysis permitted comparison of the LMC tier 1 award holders with the LMC tier 3 award holders. The LMCs with tier 1 awards came from only six medical schools: Aberdeen, Edinburgh, London, Oxford, Sheffield and Southampton. In contrast, the tier 3 award holders came from a more diverse background of 13 medical schools: Aberdeen, Cambridge, (University College) Dublin, Edinburgh, Glasgow, RCS Ireland, Leeds, London, Manchester, Mysore (Medical College), Nottingham, Oxford and Sheffield.

Table 3 compares the continental locations of medical schools of origin of LMC and non-LMC merit award holders for the ten medical schools with the greatest numbers of award holders. 97.9% of LMC merit award holders were from European medical schools, whereas 90.9% of the non-LMC award holders were from European medical schools. Here Pearson’s Chi-Square test showed no statistically significant difference between the continental locations of the medical schools of origin for LMCs and non-LMCs, p > 0.05.

5.3% of the LMC merit award holders were international medical graduates (IMG), whereas 12.1% of the non-LMC merit award holders were IMGs. *The IMGs showed the greatest representation in the tier 3 category of merit awards, in fact 100% of the IMG LMC award holders were in tier 3 where they comprised 6.9% of the tier 3 LMC awards. In comparison IMGs accounted for 13.2% of the non-LMC award-winners in tier 3.* Considering both LMC and non-LMC award holders together, the international medical graduates were 11.4% of the total.

**Table 1:** Top 10 medical schools; analysis by number of LMC award holders and number of non-LMC award holders as of 2020

Medical school	Total number of award holders	Number of LMC award holders	Percentage of LMC award holders	Number of non-LMC award holders	Percentage of non-LMC award holders
London	179	14	14.74	165	20.52
Glasgow	113	15	15.79	98	12.19
Edinburgh	84	10	10.53	74	9.20
Aberdeen	60	18	18.95	42	5.22
Oxford	45	8	8.42	37	4.60
Cambridge	43	5	5.26	38	4.73
Manchester	38	5	5.26	33	4.10
Birmingham	29	2	2.11	27	3.36
Dundee	29	0	0.00	29	3.61
Nottingham	26	1	1.05	25	3.11

**Table 2:** Top 10 medical school rankings by number of graduates holding merit awards; with or without approximate size correction as of 2020

Medical school	Total number of LMC award holders	Ranking by number of LMC award holders	LMC ranking of medical schools after size correction	Total number of non-LMC award holders	Ranking by number of non-LMC award holders	Non-LMC Ranking of medical schools after size correction
Aberdeen	18	1	1	42	4	5
Glasgow	15	2	2	98	2	1
London	14	3	7	165	1	7
Edinburgh	10	4	4	74	3	2
Oxford	8	5	3	37	6	3
Cambridge	5	6	5	38	5	6
Manchester	5	7	6	33	7	8
Birmingham	2	8	8	27	9	10
Nottingham	1	9	9	25	10	9
Dundee	0	10	10	29	8	4

**Table 3:** A geographical comparison of the medical schools of origin of LMC and non-LMC merit award holders, as of 2020

Continental location of medical school	Non-LMC			LMC	
	Total number of non-LMC award holders	Percentage of total number of non-LMC award holders		Total number of LMC award holders	Percentage of total number of LMC award holders
Europe	731	90.9		93	97.9
Asia	39	4.85		2	2.11
Africa	19	2.36		0	0.00
North America	5	0.62		0	0.00
Australasia	9	1.12		0	0.00
South America	1	0.12		0	0.00
Total	804	100%		95	100%

## Discussion

### LMC merit awards and UK medical schools

This study is the first comprehensive peer-reviewed analysis of British merit award holders’ medical schools of origin; focusing on LMCs versus non-LMCs. It serves to identify university medical schools contributing to the outcome of excellence in the medical education [19] of doctors who have subsequently become award-winning clinicians. Naturally, the information we provide will be of importance to current and future graduates from International Medical Programs [20] as well as local prospective medical students.

The 2019 General Medical Council workforce study confirms that the UK is a significant career destination for international medical graduates, [21] in fact it was stated that “For the first time, more non-UK medical graduates took up a licence to practise than UK medical graduates.“ Accordingly, the pool of possible medical schools of origin of the award-winners has essentially become worldwide. In our database of 2019 award-winners, 85 medical schools were represented.

Our results demonstrate that after being selected through a transparent and defensible assessing and scoring arrangement [22] for merit award applicants, the majority of LMC merit award holders originate from a handful of medical schools. 68.4% of the LMC award holders came from only five British medical schools (Table 1). Specifically, these were London, Glasgow, Edinburgh, Aberdeen and Oxford university medical schools. A similar result was noted amongst the award holders from all the other specialties combined (non-LMC) in the 2019–2020 round. 56.5% of all non-LMC merit award holders came from only six British medical schools. Specifically, these were London, Glasgow, Edinburgh, Aberdeen, Oxford and Cambridge university medical schools. The fact that both the LMC, non-LMC and pooled award holder rankings show such a disproportionate concentration of merit award winners in similarly small groups of medical schools, implies that together with the demonstrated excellence of these award-winning doctors, there is also structural bias at some point in the merit award schemes’ application, assessment or allocation processes. This quantitative description of bias is also likely to have been noted empirically and anecdotally by both the applicants and assessors during the more than 60 years that the award schemes have been in place. Regrettably, this would directly affect the medical community’s perception of the award schemes. Such prestigious, lucrative and longstanding schemes should be seen to be beyond reproach with respect to parity and equality when dealing with doctors of diverse educational backgrounds.

As the top ten medical schools of origin for the LMC award-winners include London, Oxford and Cambridge then in this instance the prestige and good quality of medical education appear to coincide in these universities. [23] In contrast, the high ranking of Aberdeen medical school amongst LMC award-winners implies that a prestigious medical school alone is not necessarily the dominant a factor in the successful career development of LMCs. Our new rankings of medical schools provide information by which prospective students can choose appropriate medical schools; it is recognized that students make rational decisions in the realm of education [24, 25] and information of this type is particularly relevant to a career pathway as complex as medical training. Such guidance is likely to have valuable longevity, as recent studies have shown that the differences in medical education between medical schools remain stable over time. [26].

Aside from the high quality of their medical training, the apparent numerical overrepresentation of award-winning doctors who are graduates of a small number of university medical schools probably also reflect additional contributions due to the considerations below, either individually or in combination:

1) London medical schools combine to be one of the largest university medical schools in Europe when judged by number of yearly graduates. So, in proportion, London university is likely to be well represented in any apparently Eurocentric merit award schemes. To explore such an effect we performed an approximate size correction to the medical school rankings by number of award holders, using the 2019 student admission numbers. Considering LMC, prior to the size correction London university medical schools ranked 3 but dropped to 7 after the correction (Table 2). Accordingly, a contribution to the rankings by medical school size would appear important, however, it is not clear that that size alone can account for the concentration of award holders in a handful of medical schools. Obviously, the high quality of undergraduate education in some medical schools must be an important factor in these rankings.

2) The reputations of these top medical schools and their graduates may be either implicitly or explicitly disproportionately influencing the merit award selection processes. However, this factor does not preclude educational excellence being contributory to award-winning.

3) The overrepresented medical schools have had many years to attract and retain educators (possible award-winners themselves) who can give their students the best possible advantage in their post qualification careers. Essentially, these medical schools are likely to also have acquired the expertise to prepare their medical students for the merit award steeplechase. Being made at least implicitly aware of the expected attributes and achievements of merit award holders whilst still in medical school would provide these students with more time to attain such advantageous goals.

4) The international language of medicine is English and the assessment of the applications for these merit awards is also performed in English. Naturally, this would tend to favour applications from graduates of British medical schools. It could also be argued that the more traditional British university medical schools that require a more demanding use of written English would also tend to be more successful under the current merit award schemes.

### LMC merit awards and international medical schools

The medical schools of origin of award holders were also analyzed by continental location, this being pertinent to the travel and relocation of medical professionals in the modern era of globalization. [27] This geographical diversity is also a good proxy for diversity of nationality amongst the merit award holders. For example, 99.4% of US medical students are American and 92.5% of UK medical students are UK natives (the number of international medical students that can be accepted by a medical school is capped at 7.5% by the government). Similarly, the vast majority of European medical graduates would be European natives, the vast majority of Asian medical graduates would be Asian natives etc. Accordingly, the continental medical schools of origin of the LMCs and non-LMCs merit award holders were compared (Table 3). The vast majority of LMC and non-LMC merit award holders were trained in European medical schools (97.9% and 90.9%, respectively). A Chi-square test compared the continental distributions of merit award holders and showed that there was no statistically significant difference between the LMCs and non-LMCs, p > 0.05.

Future analyses of the medical schools of origin by country of LMC award holders may be more illuminating as the greater numbers and greater diversity of international medical graduates allow for more meaningful statistical calculations. Currently, the numbers are too small for productive analysis by country of medical training.

An unexpected finding of this study was the greater diversity of the medical school origin amongst the lowest grade of national merit award holders than the highest grade of national merit award holders. Specifically, the data show that all the tier 1 LMCs came from just 6 medical schools: Aberdeen, London, Oxford, Edinburgh, Sheffield and Southampton medical schools. In contrast, the lowest national tier of award holders (tier 3) came from a more diverse background of 13 medical schools: Aberdeen, Cambridge, (University College) Dublin, Edinburgh, Glasgow, RCS Ireland, Leeds, London, Manchester, Mysore (Medical College), Nottingham, Oxford and Sheffield. The latter list of 13 medical schools includes an Indian medical school, as well as including more than twice as many medical schools as the top LMC award holders. Not only are the number of schools more diverse amongst the tier 3 award holders but there is also the greatest proportion of international medical graduates in tier 3. Specifically, considering the tier 3 LMCs, 5.3% of the total were IMGs and *all the IMG award holders were in tier 3*. There were no tier 1 or tier 2 IMG award holders. Similarly, the non-LMC IMG award holders were concentrated in tier 3. This change may reflect recent globalization trends that are increasing the number of IMGs working in the UK as well as a permissive inclusivity in these lower national merit award allocations. In time, such a trend may also be reflected in the higher awards. If this change is significant and the allocations are impartial, longitudinal analyses of merit award holders over the next decade would be valuable in accurately assessing whether this IMG trend extends to the tier 1 merit awards.

### Undergraduate training, postgraduate training and LMC merit awards

As a result of the original nature of our study we were unable to identify other studies that examined the effect of UK medical school training on subsequent merit award-winning doctors. However, there were a small number of studies that analyzed the variation in UK medical school performance and related this to early postgraduate career performance. [26, 28, 29] The most comprehensive was the 2020 study (*MedDifs*) by McManus et al. which examined the differences in medical school performance using 50 measures, both quantitively and qualitatively, that were divided into the categories of institutional history, curricular influences, selection of applicants, teaching/learning and assessment, student satisfaction, foundation entry scores, F1 perception, specialty training choice, fitness to practice and postgraduate examination performance. In comparison, our study was limited in not being as granular in the number of measures used and in not making use of a qualitative approach. The MedDifs study was able to observe the relationships between the large number of measures (e.g. PBL schools producing lower performance in postgraduate exams, graduates of smaller medical schools performing better in postgraduate exams, graduates of schools with more self-regulated learning performing better in postgraduate exams and variation in the likelihood of graduates from specific schools entering particular specialties) but was less able to describe the causal relationships. Both their study and this study had the limitation of being unable to comparatively analyze the different medical schools at the level of courses within the schools.

To shed light on the possible causality of our medical school rankings we examined the history of the UK medical schools. [30–39] We observed that all 7 of the oldest medical schools in the UK were represented in the top 10 medical school rankings of our LMC and non-LMC award holders. Specifically, these medical schools were all formally established before 1826; Edinburgh in 1726, St George’s London University in 1733, Glasgow in 1751, St Bartholomew’s London University in 1785, Aberdeen in 1786, Manchester in 1824 and Birmingham in 1825. Interestingly, Oxford medical school had been teaching medicine since the 12th century and teaching medicine in Cambridge had been occurring since at least 1524; effectively these two medical schools were informally established before a formal establishment process existed. With this in mind, of the top 10 medical school rankings for LMCs and non-LMCs, *8 are the oldest medical schools in the UK.* Furthermore, none of the more modern medical schools (established in or after the year 2000) are represented in our top 10 medical school merit award rankings for LMCs or non-LMCs. Specifically, Warwick in 2000, Norwich in 2000, Peninsula in 2000, Brighton and Sussex in 2002, Hull York in 2003, Keele in 2003 and Swansea in 2004 are not yet represented in the top 10 merit award rankings. Whilst it understandable that the medical schools established within the last decade may not have had time to distinguish themselves at the merit award level, it is less clear that this is true for the medical schools established around the year 2000.

The totality of these observations is consistent with at least a correlation between medical school age and number of alumni holding merit awards. Considering the results of our study and also accepting the results of the studies into UK medical school education, [26, 28, 29] we propose a model describing medical school performance that is consistent with the currently available data:

**A model of excellence in medical education; a cycle of institutional memory and experience**.

1) The **older** medical schools have **more institutional memory and experience** in education. As a result of longevity they are more likely to have produced some successful graduates.

2) The more **able and ambitious students** are then more likely to be **attracted** to these medical schools which are perceived to have produced successful alumni, and so apparently have **better reputations** and appear more prestigious.

3) These medical schools with the **greater institutional memory and experience** in education are better placed to use this background knowledge to more support and **facilitate better education and better educators**.

4) Consequently, these medical schools will then have both a greater concentration of **more able students and more able educators**.

5) These students in these institutions are then more likely to experience **higher quality teaching**, better **mentoring** and better **career advice**.

6) Subsequently, these institutions are **more likely** to produce graduates who become **clinically excellent award-winners**. Having experience of creating these successful doctors will **add to** the institutional memory and experience in education of these medical schools and **the cycle will repeat**.

It should be noted that the older medical schools have had more time to undergo more repeats of this cycle, creating a cumulative effect and accordingly increasing the number of clinically successful and excellent award winners originating in their schools. We suggest that part of the reason for the differences between medical school educational performance may relate to the relative effectiveness of the cycle in different medical schools.

It should also be noted that the same conditions that apply to the development of this cycle of institutional memory and experience, also apply at the college/departmental level. In the case of LMCs, a college or department that produces good LMCs is more likely to produce more award-winning LMCs. Essentially, this would be a cycle of *departmental* memory and experience.

We are aware that this proposed cycle will have positive effects on postgraduate training as well. The award-winning celebrated alumni of these medical schools are more likely to be perceived as leaders in medicine, inspirational figures and contribute to more respected postgraduate mentorship.

This model is also helpful in addressing the apparent concentration of merit award holders from particular medical schools - the effect of bias. With each cycle of the model greater numbers of successful graduates from the older medical schools accumulate in the medical community. These alumni will become more visible professionally and are also more likely to acquire influential senior management or administrative positions, such as merit award allocators. From that point, implicit or explicit selection bias effects will favour the older medical school alumni in award allocation. We believe this model wholly or partly explains the apparent confluence of both bias and excellence in our medical school award-winners rankings. It seems plausible that the two effects are linked and are likely to occur together.

In January 2022 the UK government announced an update to the Clinical Excellence Award scheme, to be called the National Clinical Impact Awards. [40] The stated aims of the new scheme were to (1) broaden access, (2) make the application process simpler, fairer and more inclusive, and (3) ensure the scheme rewards and incentivises excellence across a broader range of work and behaviours. [41] If our institutional memory and experience model has value, we would anticipate that an analysis of this latest iteration of a national merit award scheme will yield similar rankings to those demonstrated in our study.

We can indicate that our analysis of the merit awards is designed to focus on the medical school of origin as an important factor contributing to subsequent clinical excellence. We limited our study to this rarely investigated factor. We entirely accept that many other factors also play roles in the both subsequent clinical excellence and in award allocations. Obviously, gender, age, ethnicity, regionality, teaching or general hospital hospitals will all play roles in award allocation and these factors have been previously studied and have been extensively discussed. [1–13] Future studies would benefit from an examination of the intersectionality of these factors and the medical schools of origin with a view to the final merit award allocations. Experienced educators are intuitively aware that the success of any medical trainee is a multifactorial equation that we try to influence in as positive a manner as possible. Our data support the observation that the medical school of origin remains an important factor as a potential predictor of medical career success, long after the formal university education is complete. As such, this is a factor that students, trainee interviewers and medical educators should consider in their decision making.

## Conclusions

Using clinical excellence awards as an outcome measure, our study adds original medical education data to the pool of information that describes the demographic distribution of clinical excellence in Britain. We show that both educational excellence and bias towards some of medical schools are demonstrated amongst merit award-winners. Specifically, we identify the medical schools that are most associated with the production of excellent award-winning laboratory medical doctors. We identify the medical schools that are most associated with the production of excellent award-winning non-laboratory medical doctors. *We are the first to produce a ranking of medical schools by the number of excellence award-winners.* We indicate the importance of these medical school rankings to prospective students and educators. We proffer an original model to explain our medical school rankings that may have a wider applicability.

We show that international medical graduates are beginning to make a significant contribution to LMC and non-LMC clinical excellence in Britain, particularly amongst the lower national merit awards.

.

## Data Availability

data from this article is available upon reasonable request to the authors. Dr Steele is the corresponding author will make the data available. https://www.sehd.scot.nhs.uk/publications/DC20200319SACDA.pdfhttps://www.gov.uk/government/publications/accea-annual-report-2020https://www.gmc-uk.org/registration-and-licensing/the-medical-registerhttps://olr.gdc-uk.org/SearchRegister.

## Declarations

### Authors’ information

Both authors are affiliated to Ajman University of Science and Technology.
